# The superoxide dismutase (SOD) genes family mediates the response of *Nilaparvata lugens* to jinggangmycin and sugar

**DOI:** 10.3389/fphys.2023.1197395

**Published:** 2023-05-10

**Authors:** Ahmad Sheraz, Haowen Zhu, Qiaoqiao Dong, Tingting Wang, Suman Zong, Huaiqi Wang, Linquan Ge, Tao Wu

**Affiliations:** ^1^ College of Plant Protection, Yangzhou University, Yangzhou, China; ^2^ College of Horticulture and Landscape Architecture, Yangzhou University, Yangzhou, China

**Keywords:** superoxide dismutase, *Nilaparvata lugens*, jinggangmycin, glucose, sucrose, trehalose, bioinformatics

## Abstract

**Introduction:** Brown planthopper (BPH), *Nilaparvata lugens* Stål (Hemiptera: Delphacidae), is a major rice pest causing significant damage to rice throughout the world. Intensive pesticide usage often causes resistance in these seasonal pests, mainly through the modulation of antioxidant machinery. The superoxide dismutase (SOD) gene family is known for regulating BPH response to pesticides.

**Methods:** In the present study, we identified eight *NlSOD* genes from the NCBI using the BLASTP program. The bioinformatics analysis includes a phylogenetic tree, conserved domain, motifs, gene ontology (GO) analysis, Kyoto encyclopedia of genes and genomes (KEGG) pathways, and protein-protein interaction, highlighting the distinctive functional elements of *NlSOD* genes.

**Results and discussion:** Additionally, the *NlSOD* genes showed expression in all developmental stages of BPH. Under three sugars (glucose, sucrose, and trehalose) treatment, the respective upregulation of *NlSOD8, NlSOD6,* and *NlSOD2* was noted. The *NlSOD1* induced significantly under jinggamycin (JGM) deduced its potential as a key regulator of BPH response to the pesticide. Our study has provided detailed knowledge of the *NlSOD* gene family in-silico analysis and the defensive response to insecticide and high sugar of BPH. We hope the results of this research will help to shed light on the resistance of BPH towards insecticide toxicity and high sugar and help to control it more efficiently.

## 1 Introduction

Aerobic organisms thrive on oxygen through mitochondrial machinery in order to sustain life. Some of these oxygen molecules are converted into reactive oxygen species (ROS) during metabolic processes (Atalay et al., 2020). Exposure of tissues and cells to radiation (ultraviolet rays) and chemical agents also produces ROS (Lee et al., 2019; Qureshi et al., 2021b). ROS, including superoxide anion (O_2_^−^) and hydrogen peroxide (H_2_O_2_), react nonspecifically with various substances and damage cells (Hermes-Lima and Zenteno-Savín, 2002; Fukai and Ushio-Fukai, 2011). Aerobic organisms have antioxidant enzymes that scavenge or remove ROS to prevent such oxidative damage. SOD (EC 1.15.1.1) is one such antioxidant enzyme that catalyzes the dismutation of superoxide anions into molecular O_2_ and H_2_O_2_. Thus, SOD breaks down superoxide anions generated in cells. It plays an important role in protecting organisms from oxidative damage (Valentine and Hart, 2003a; Fukai and Ushio-Fukai, 2011). SOD is a metalloenzyme constituting three types: cytosolic Cu and Zn-containing (Cu/Zn-SOD), mitochondrial Mn-containing (Mn-SOD), and extracellular (Ec-SOD) SOD (Okado and Fridovich, 2001; Yamamoto et al., 2005).

The *SOD* gene family provides the first line of defense against ROS primarily generated by aerobic metabolism (Parker et al., 2004). SOD enzymes transform negatively charged oxygen molecules into hydrogen peroxide, which catalase then destroys. Not only are ROS extremely harmful to cellular components, but research is increasingly identifying ROS as one of the primary mediators of aging processes (Sohal et al., 2002; Hasty et al., 2003; Sharif et al., 2022). This protective role of SOD is prevalent in both eukaryotic and prokaryotic organisms (Sharif et al., 2022). Thus, it’s quite possible that some major *SOD* gene families are also implicated in insect body plans (Bordo et al., 1994; Zelko et al., 2002).

There are three basic families of animal SOD enzymes with two possible metal configurations in the active sites using either Mn or Cu and Zn ions (Bordo et al., 1994). The Mn-SOD is restricted to the inner matrix of the mitochondria (Okado-Matsumoto and Fridovich, 2001) and is very different in structure from the Cu/Zn-SOD. Of the two Cu/Zn-SOD, one gene product is primarily localized in the cytoplasm (Crapo et al., 1992), with some also occurring in the mitochondrial intermembrane space (Okado-Matsumoto and Fridovich, 2001). This gene is usually referred to as the cytoplasmic Cu/Zn-SOD. The Cu/Zn-SOD is predominantly extracellular, although it has also been shown to be membrane-bound (Folz et al., 1997; Fujii et al., 1998). When this second SOD, termed extracellular Cu/Zn-SOD, is secreted rather than routed to the membrane, it can be reabsorbed and transported inside the nucleus, where it protects genomic DNA (Ookawara et al., 2002) and slows telomere shortening (Von Zglinicki, 2002; Serra et al., 2003). The extracellular Cu/Zn-SOD gene is distinguished from the cytoplasmic Cu/Zn-*SOD* gene by having an N-terminal signal cleavage peptide that routes the extracellular Cu/Zn-*SOD* for secretion (Folz et al., 1997; Fujii et al., 1998).

In recent decades, rice consumption has increased globally. A statistical analysis of rice consumption showed a high increase. In the 2008/2009 crop year, 437.18 million metric tons (MMTs) of rice were consumed; compared to 2021/2022, 509.87 MMTs worldwide (Shahbandeh, 2021). Although the rice crop gets affected by a number of insect pests, however, BPH is one of the most destructive insects of rice (Hu et al., 2014). Rice is directly affected by BPH infestations, which also disseminate several viral pathogens, such as the rice grassy stunt virus (RGSV) and rice black-streaked dwarf virus (RBSDV) (Zhou et al., 2008; Nguyen et al., 2015). Host plant resistance is the best approach for mitigating BPH; however, pesticide measures are at the top of the list because of their broad availability and expeditious results (Jena et al., 2006). JGM is a fungicide utilized for the treatment of rice fields for the disease *Rhizoctonia solani*, which leads to rice sheath blight (Jiang et al., 2012). BPH’s flight ability, body weight, thermo-tolerance, protein and lipid contents, and reproduction are all reportedly impacted by the JGM (Ge et al., 2019; Ahmad et al., 2022b).

The pesticide applications on pest population notoriety remain an important research area. The JGM fungicide, a product of *Streptomyces* var. jinggangen, is used for controlling rice sheath blight *R. solani* commercially used in BPH-threatened areas in China (Peng et al., 2014). JGM is commonly applied two to three times in the rice growing season at a commercial level of 125–175 g.a.i. ha^-1^ through the foliar spraying method. As experienced with insecticides, the JGM sprayed leaf enhances the BPH survival rate and fecundity (Wu et al., 2001; Jiang et al., 2012). JGM is generally overused due to its low toxicity and low impact on biodiversity. SOD studies in insects are comparatively rare compared to plants and mammals. However, some of the genes have been studied in insects, such as *SOD1* in *B. mori* (Yamamoto et al., 2005a) and *Hyphantria cunea* (Yamamoto et al., 2007), as well as *SOD2* in *B. mori* and *NlSOD1* in *N. lugens* (Yamamoto and Yamaguchi, 2022). In the current study, we have systematically characterized the *NlSOD* gene family in BPH. Further, the gene structure, conserved motifs, protein-protein interactions, KEGG enrichment pathways, GO analysis, and the expression analysis of *SOD* genes in response to high sugar treatment and JGM have been studied. We believe our work will contribute to future functional studies regarding pesticide resistance in BPH and minimize the overuse of toxic pesticides on rice crops.

## 2 Materials and methods

### 2.1 Identification of *NLSOD* gene family members in *N. lugens*

To obtain insights into the *NlSOD* gene family, the protein sequences of *N. lugens* were retrieved from the publically available National Center for Biotechnology (NCBI) (http://www.ncbi.nlm.nih.gov/cdd/) and eight *NlSOD* genes were identified. For the phylogenetic relationship of the *NlSOD* gene family, 8 NlSOD, *Drosophila melanogaster* (3 DmSOD), *Laodelphax striatellus* (3 LsSOD), *Scirpophaga incertulas* (4 SiSOD), *Spodoptera frugiperda* (4 SfSOD) proteins sequences were downloaded from the NCBI online database. Furthermore, To avoid the possible loss of a single NlSOD protein due to the fact of a missing domain, a local BLASTP with a 1E-5 cutoff was performed. The Conserved Domain Database (CDD) of the NCBI (http://www.ncbi.nlm.nih.gov/cdd/) (accessed on 14 January 2023), SMART database (http://smart.embl-heidelberg.de/) (accessed on 18 January 2023) (Schultz et al., 1998), Inter Pro Scan program (https://www.ebi.ac.uk/interpro/) (accessed on 18 January 2023), and Scan Prosite (https://prosite.expasy.org/scanprosite/) (accessed on 20 January 2023) was used to confirm the *NlSOD* gene family-specific domains. Additionally, the physiochemical properties of the NlSOD proteins in the studied species were discovered using the ExPASy online server (http://web.expasy.org/protparam/) following the same protocol as the previous study of Sohail et al. (2022).

### 2.2 *NlSOD* gene family phylogenetic relationship, conserved domain, motif distribution, and GO analysis

To obtain detailed knowledge regarding the evolutionary relationship of the *NlSOD* gene family in *N. lugens* with the counter species. Herein, we investigated the phylogenetic relationships of the *NlSOD* gene family members of *N. lugens* with the developed genome *D. melanogaster* and the other three insects species (*L. striatellus, S. incertulas,* and *S. frugiperda*). Firstly, all the mentioned insect’s SOD amino acid sequences were downloaded from NCBI and were then aligned through ClustalW software (version 2.1) (http://www.genome.jp/tools/clustalw/) (accessed on 13 February 2023) following the default parameters to examine the evolutionary relationships among the sequences and construct the maximum likelihood (ML) phylogenetic tree using MEGA7 (version 7.0) (Kumar et al., 2016). For the reliability of clades, bootstrap with 1,000 replicates was used.

Domain architecture of identified NlSOD proteins was performed by using the full-length protein sequence to CDD NCBI (https://www.ncbi.nlm.nih.gov/Structure/cdd/wrpsb.cgi) (accessed on 23 February 2023) and downloaded hit data subjected to DOG software (version 2.0) (visualization of protein domain structure) for domains visualization and finally for better graphical representation the Microsoft PowerPoint 365 was used.

Furthermore, the conserved motifs of the *NlSOD* gene family were predicted using the MEME online server (Version 4.12.0) (http://meme-suite.org/) (accessed on 23 February 2023) with the default settings. The details of the top 5 predicted motifs were obtained from the MEME suite and then cross-checked with NCBI-CDD (http://www.ncbi.nlm.nih.gov/Structure/cdd/wrpsb.cgi) (accessed on 23 February 2023). Finally, the motif distribution was drawn via Microsoft PowerPoint 365 software.

The obtained NlSOD protein sequences were implied in the online server “CELLO2GO” (http://cello.life.nctu.edu.tw/cello2go/alignment.php) to determine the predicted functions, such as molecular functions, biological processes, and cellular components. Finally, the GO classifications were recovered using GraphPad Prism (Version 9.4.1) (GraphPad Software, Inc., LA Jolla, CA, United States) was used for graphical representation.

### 2.3 Protein interaction network construction

The online server String (version 11.5) (https://string-db.org) designed by the Swiss Institute of Bioinformatics (accessed on 25 February 2023) was used for the protein-protein interaction (PPI), and the network was recovered using the NlSOD1 protein as our reference protein. The obtained results were based on the available sources in the string database, including text mining co-expression, determined, co-occurrence, intersection, curated databases, and fusion (Szklarczyk et al., 2015). The pathway enrichment analysis was also conducted by searching for *NlSOD* genes in the NCBI database’s online pathway enrichment tool.

### 2.4 Insect rearing, stress treatment, and expression profiling of the *NlSOD* genes

The insects BPH and the rice verity Ninjing4 were used in the study, obtained from China National Rice Research Institute (Hangzhou, China). The Ninjing4 rice variety, known to be preferred because of no resistance to BPH infestations, was used for rearing. Initially, the BPH colonies were reared on the rice seedlings in cement tanks covered with fine mesh outdoors (i.e., in natural conditions) for 6 months (i.e., April to October) and overwintered in lab-controlled conditions.

First, we soaked the seeds for 24 h in a water dip plastic tray with a standard size of one-quarter (60 cm H_100 cm W_200 cm L) in lab standard conditions of 26°C ± 2°C with the 16 h L:8 h D in relative humidity of 80% ± 10% in the ecological laboratory of Yangzhou University. The germinated seeds were transferred to cement tanks covered with fine mesh in an outdoor natural environment and were grown until the six-leaf seedling stage. The seedlings were then transferred into plastic pots (dimensions = R ¼ 16 cm). Finally, the stress treatments proceeded at the tillering stage (40 ± 2, 40 ± 4, and 40 ± 8 days) following the same procedure as Ahmad et al. (2022a). Furthermore, the JGM technical grade of 61.7% used in this study was obtained from the Qianjiang Biochemistry Co., Ltd. (Haining, Zhejiang, China). Following the protocol in a previous report by Ahmad et al. (2022a), the two hundred parts per million (PPM) solution was Tween 20 obtained from the Sinopsin Group Chemical Reagent Company (Shanghai, China). The fungicide was then sprayed on the rice seedlings, following the procedure in a previous study (Ge et al., 2010).

The rice seeds were sown in standard cement tanks containing standard rice-growing soil at the Yangzhou University experimental field under natural outdoor conditions (Ge et al., 2013). Seedlings bearing six leaves were transplanted into plastic pots (R ¼ 8 cm) with four hills per pot, three plants per hill, and grown under natural conditions for experiments. Rice plants were pulled from the pots (at about 25 days), keeping the root system intact. The plants were gently washed without damaging the roots, then cultured in plastic containers (D ¼ 12 cm and H ¼ 25 cm) containing rice hydroponic liquid solution obtained from International Rice Research Institute (Los Banos, Laguna, Philippines). The pH of the hydroponic solution was maintained at 5.0 by daily adjustments with 1 mol/L HCl and 1 mol/L NaOH. The root systems were immersed in the hydroponic solution, and the solution was replaced every 3 days to ensure sufficient plant nutrition. Experimental rice plants (n ¼ 3) were transferred into a 1% glucose, sucrose, and trehalose hydroponic solution after a week following the method of Lin et al. (2018), and controls (n ¼ 3) were cultured in normal hydroponic solution (without sugar). Thirty-third-instar nymphs were transferred onto rice plants in the 1% glucose, sucrose, and trehalose hydroponic solution and covered with 60 mesh nylon net, and the BPH nymphs infestations for two, four and 8 days were collected and snap frozen in liquid nitrogen then and stored in −80°C degree till further use. Finally, the stored samples were then used for qRT-PCR analysis, and the specific primers used in the study are listed in (Supplementary Table S1).

### 2.5 Expression profiling of the *NlSOD* genes in *N. lugens*

BPH was introduced on the forty-day-old (40 ± 2) to plastic buckets having rice plants, and samples were collected at 2, 4, and 8 days after infestation. First, the BPH samples were snap-frozen in liquid nitrogen and then stored at −80°C degrees till further experiments. Furthermore, total RNA was extracted from the samples using an RNA extraction kit (Vazyme, Nanjing, China). First, the free DNA was removed using DNase I, and the concentration and purity were measured using a NanoDrop 1,000 spectrophotometer (Thermo Fisher Scientific, Rockford, IL, United States). Also, the integrity was checked using 1.5% agarose gel electrophoresis. Finally, the resulting complementary DNA (cDNA) was used as a template for quantitative real-time PCR (qRT-PCR) analysis using SYBR Green real-time PCR master mix (Vazyme, Nanjing, China). The qRT-PCR assays were performed in triplicate using a real-time PCR system (Bio-Rad, Hercules, CA, United States) following the manufacturer’s protocol.

Furthermore, the 2 μL aliquots of cDNA were amplified by qPCR in 20 μL reaction volumes using the SYBR Premix Ex Taq™ II (TaKaRa, Dalian, China). The cDNAs were amplified at 95°C for 2 min, followed by 35 cycles of 10 s at 95°C, then for 30 s, and at 72°C for 30 s, with a final extension step of 72°C for 10 min in a CFX96 real-time PCR system (Bio-Rad Co., Ltd., CA, United States). The mRNA amounts of all genes were separately quantified with the stable expression of the constitutive reference gene, actin. The specific primers are listed in (Supplementary Table S1). After amplification, the target gene cycle threshold (Ct) values were normalized to the reference gene (actin) by the 2^−ΔΔCT^ method (Livak and Schmittgen, 2001). The data’s mean values of three biologically independent replicates were used for the final graphs.

### 2.6 Statistical analysis

The data presented in this paper were analyzed using the SPSS software (version 25.0, SPSS Inc., Chicago, IL, United States) for statistical analysis (ANOVA), statistical significance, and a 95% confidence interval (*p* ≤ 0.05). The data were analyzed and expressed as the mean ± standard deviation (SD) of three biologically independent replicates in all measured parameters, and finally, GraphPad Prism (Version 9.4.1) (GraphPad Software, Inc., LA Jolla, CA, United States) was used for graphical representation.

## 3 Results

### 3.1 Identification and sequence analysis of *NlSOD* genes

The present study retrieved 8 *NlSOD* genes, and the nomenclature was given (*NlSOD1-NlSOD8*) based on the three functional domains and chromosomal positions. Among the eight *NlSOD* gene family members, the *NlSOD1* contains two functional domains, namely, Sod_Fe_N and Sod_Fe_C; the *NlSOD4* and *NlSOD7* exhibit single domains (Supplementary Figure S1). Moreover, the five members of the *SOD* genes family (*NlSOD*2, *NlSOD3, NlSOD5, NlSOD6,* and *NlSOD8*) were found with the Sod_Cu conserved domain. The accession number and annotations of the conserved domains are listed in supplementary data (Supplementary Table S2).

Following the deep scan, eight *NlSOD* genes were isolated from the NCBI online database; the detailed information is listed in Table 1. Among them, *NlSOD1* and *NlSOD4* resided in the mitochondrial, *NlSOD2* extracellular, and *NlSOD3* nuclear, whereas *NlSOD5, NlSOD6, NlSOD7* and *NlSOD8* resided in cytoplasmic. Other features of NlSOD proteins such as locus ID, chromosomal coordinates, molecular weight, chemical properties, and isoelectric point (PI) are tabulated.

**TABLE 1:** The gene and protein features of *NlSOD* Genes.

Locus ID	Gene name	Chr. Location	Start	End	AA	MW (kDa)	PI	SL
LOC111058957	*NlSOD1*	01	96224624	96234236	231	26,042.76	6.81	M
LOC111057284	*NlSOD2*	10	16972174	16974080	162	17,156.18	5.81	E
LOC111046325	*NlSOD3*	10	11,006	16,572	120	13,504.25	10.40	N
LOC120349329	*NlSOD4*	Scaffold	7312	7857	141	14,901.89	9.17	M
LOC111061280	*NlSOD5*	07	15158027	15165199	159	16,296.20	5.95	C
LOC111046325	*NlSOD6*	10	17035966	17048858	110	12,329.93	7.14	C
LOC120349329	*NlSOD7*	Scaffold	6871	7182	108	12,208.67	4.99	C
LOC111057284	*NlSOD8*	10	17202895	17205349	173	18,036.30	5.94	C

amino acids: AA, molecular weight: MW, isoelectric point: PI, subcellular location: SL, mitochondrial: M, extracellular: E, nuclear: N, cytoplasmic: C.

### 3.2 Phylogenetic relationship and motif patterns of *SOD* genes in *N. lugens*

**FIGURE 1:**
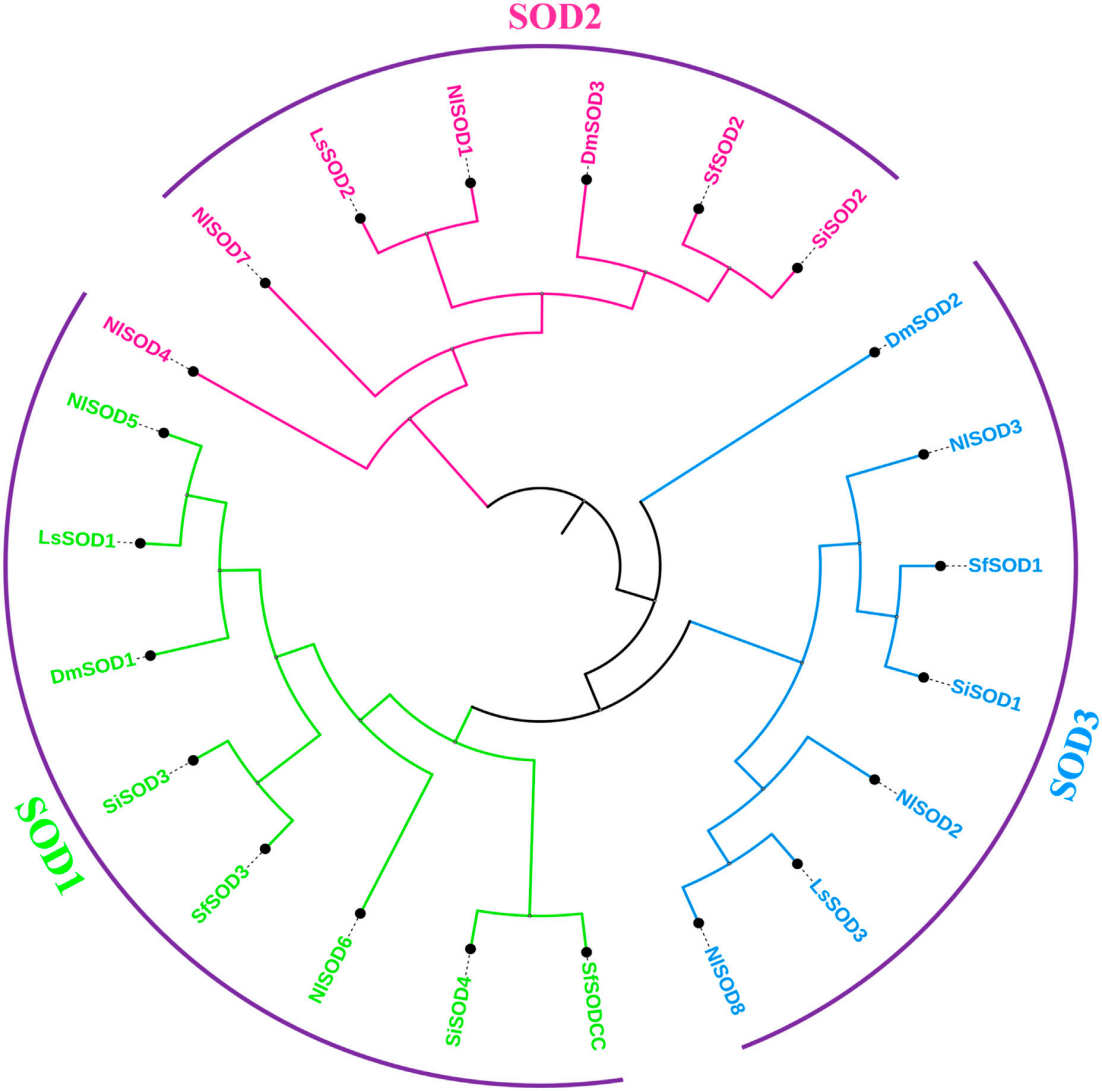
Schematic representation of the phylogenetic analysis of the *SOD* gene family: the phylogenetic tree was generated using the amino-acid sequences of selected SODs via the neighbor-joining (NJ) tree method. All *N. lugens* SODs, *D. melanogaster*, *L. striatellus*, *S. incertulas*, and *S. frugiperda*, with their counterparts, were classified into three subgroups, and the final tree was displayed using iTOL.

To obtain the evolutionary relationship of the *NlSOD* gene family, 8 NlSOD proteins with 4 other insects’ species, including 3 DmSOD, 3 LsSOD, 4 SiSOD and 4 SfSOD protein sequences were aligned using ClustalX software. After alignment, an ML phylogenetic tree was constructed using MEGA7 software (version 7.0). Finally, the phylogenetic tree was displayed using subscription-based iTOL: Interactive Tree Of Life (https://itol.embl.de/) (Letunic and Bork, 2021). The phylogenetic analysis clustered the SOD proteins into three subgroups such as SODI, SODII, and SODIII, and the number of SOD proteins was counted in each subgroup, whereas subgroup I accounted for 2 proteins and subgroup II and subgroup III with 3 NlSOD proteins (Figure 1), following the same procedure of our previous report by Ahmad et al. (2022b).

**FIGURE 2:**
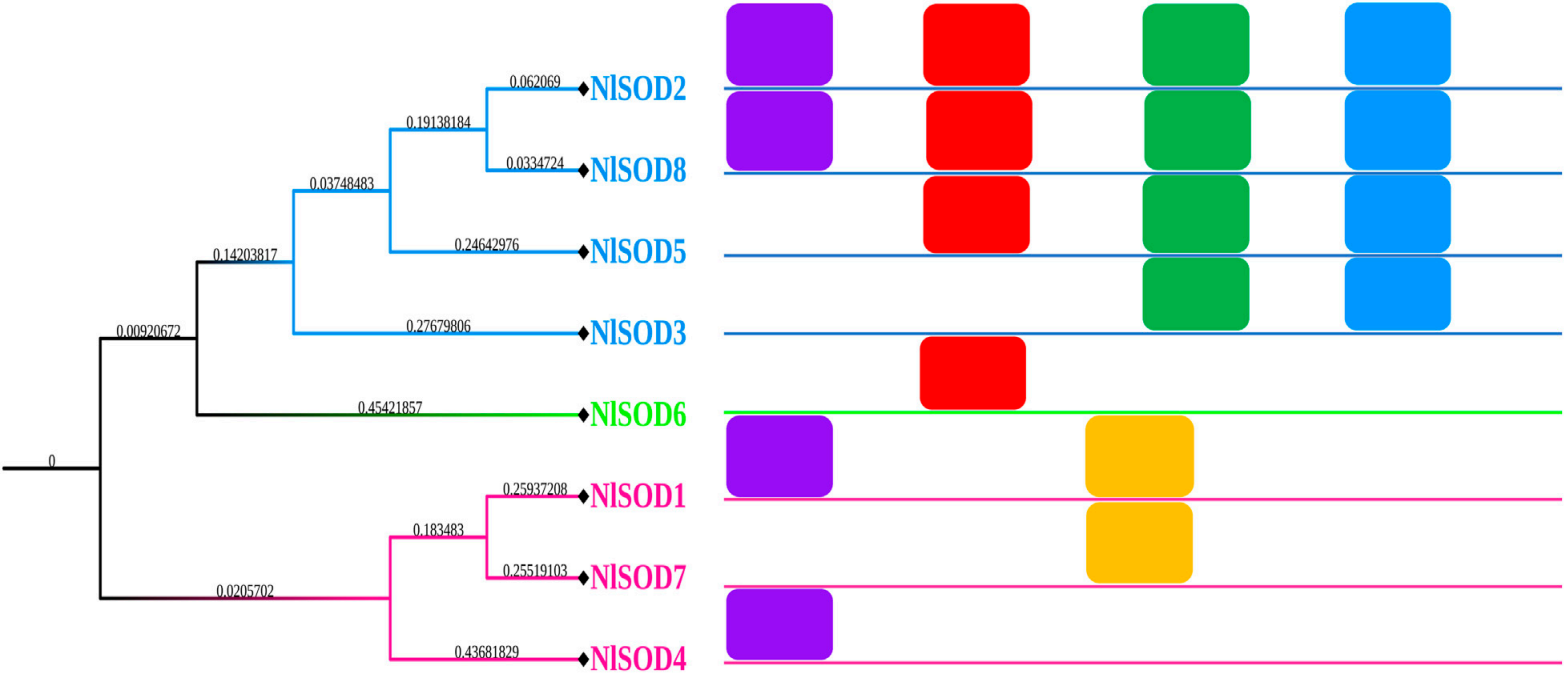
Schematic representation of the conserved motifs of *NlSOD* genes in *N. lugen.* The phylogenetic tree was recovered using Mega (V. 7.0), and then the motifs were combined using Microsoft PowerPoint 365.

A total of eighteen conserved motifs were discovered using the MEME online server (Version 5.4.1) (https://meme-suite.org/meme/tools/meme) (accessed on 26 February 2023) reported by Bailey et al. (2009), and they were found to be appropriate for explaining the *NlSOD* gene’s structure (Figure 2). Among the eight *NlSOD* genes, the *NlSOD2* and *NlSOD8* were counted with four motifs, followed by *NlSOD5* with three motifs, and *NlSOD1* and *NlSOD3* exhibited two motifs. Finally, the three *NlSOD* genes such as *NlSOD4, NlSOD6,* and *NlSOD7,* were recorded for a single motif.

### 3.3 Go analysis and KEGG pathways enrichment classification

*NlSOD* genes GO analysis showed various key functional predictions. For instance, the biological, molecular, and cellular processes following the same as our previous study by Ahmad et al. (2022b). In the biological processes category, the *NlSOD* genes are mainly involved in the regulation of stress-responsive with a high percentage of 22% and 20% in the biosynthetic process, whereas the other functions such as cell pigmentation, cell differentiation, and other environmental stimuli via hormonal and metabolic modulation were also observed (Supplementary Figure S2). As stated in the molecular processes category, the *NlSOD* genes were also found to regulate oxidoreductase activity, ion binding, protein binding, and enzyme binding activity. A large number of *NlSOD* genes reside in the intercellular region by up to 26%, followed by cytoplasm, and plasma membrane, whereas some members are localized to extracellular space and plasma membrane.

**FIGURE 3:**
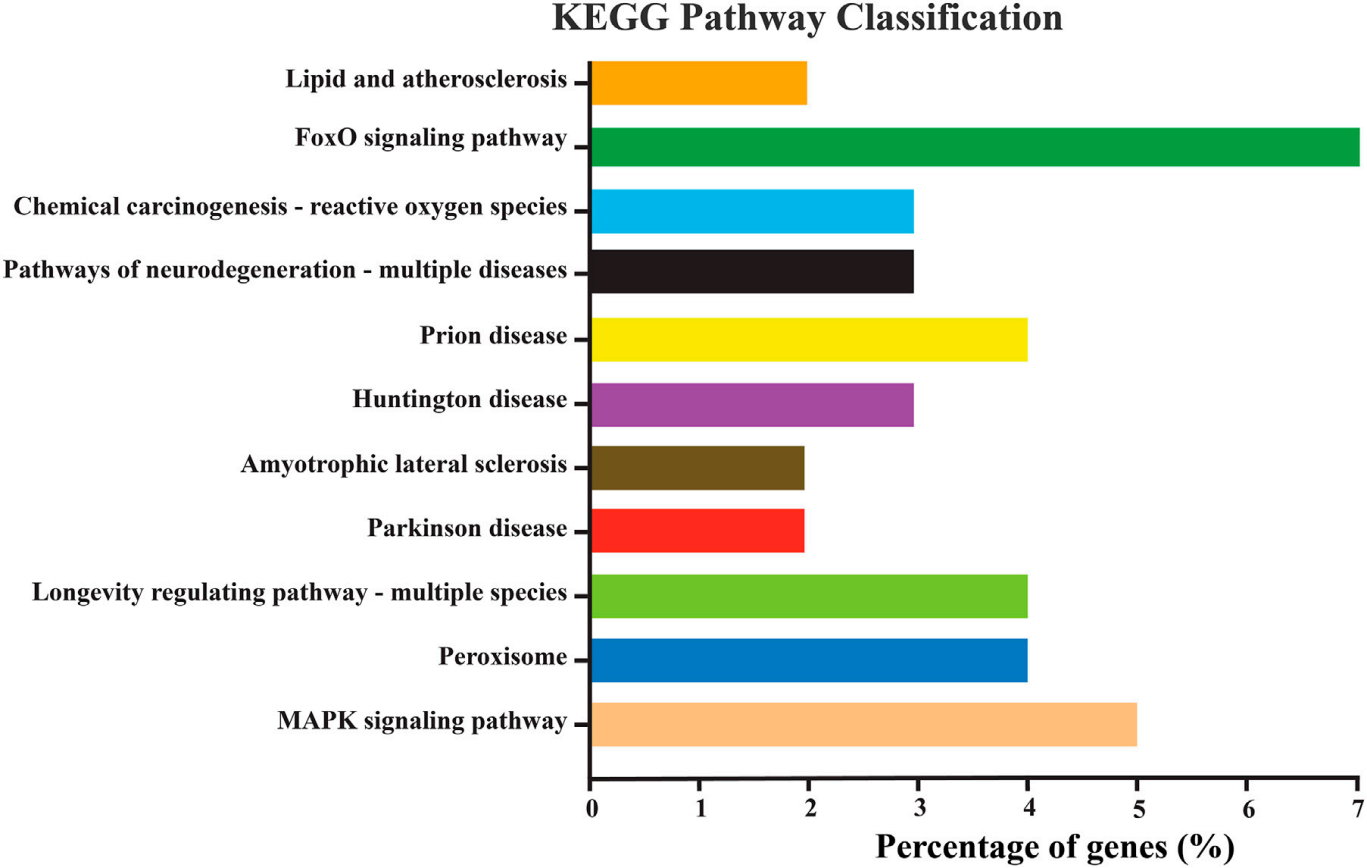
Represents the KEGG pathway enrichment analysis of differentially expressed genes of *N. lugens*. KEGG pathways with (*p-value < 0.01, log2 ≥ 5, ≤–5*). The *Y*-axis indicates the KEGG pathways, and the *X*-axis indicates the percentage of genes in each pathway. The percentage in the *X*-axis is DEGs significantly enriched in the relevant pathways.

KEGG pathway enrichment analysis is an improtant tool for understanding the molecular framework ignited by a cluster of genes. For instance, in our study, the *NlSOD* genes showed their involvement in different KEGG pathways. Amongst them, the FoxO signaling pathway was regulated by seven percent of *NlSOD* genes, followed by the MAPK signaling pathway, which accounted for five percent of genes in their regulatory machinery. The chemical carcinogenesis-reactive oxygen species signaling pathway, which is moderately influenced by three percent of *NLSOD* genes in response to JGM stress and serves as the first line of defense was also found in the analysis. Additionally, the longevity regulating pathway, which is key to insect lifespan, was also fine-tuned by *NlSOD* genes. A series of disease-related pathways such as prion, parkinson, and huntington could rely on the transcriptional of *NlSOD* genes. The KEGG pathway analysis recorded the lipid and peroxisomes pathways (Figure 3).

### 3.4 NlSOD proteins interactive network analysis

The detailed information of our reference protein NlSOD1 protein interactive partners were initially searched in the online server FlyBase (https://flybase.org/reports/FBgn0032350) (accessed on 27 February 2023) and was confirmed by STRING: functional protein association networks (https://cn.string-db.org/) and the integrative protein partners network was programmed. The detailed information and accessions are tabulated in (Supplementary Table S3). The NlSOD1 protein plays a crucial role in BPH growth and development and is also highly stress-responsive. Numerous other crucial proteins are also found to be highly interactive with our reference NlSOD1 protein. (Supplementary Figure S3). For instance, the SOD1 protein interacted highly with CAT, Prx5, PHGPx, GCLC, and CG15116. Almost similar binding was also observed for SOD2 protein with other crucial proteins such as TAM and MRPS29, which is extensively studied on the stress/diseases side. Finally, the SOD3 protein was found with high interaction with all the proteins named above, revealing the potential role of the NlSOD protein as crucial in the developmental processes and highly stress-responsive.

### 3.5 Tissue-specific expression analysis of *NlSOD* genes in developmental stages

**FIGURE 4:**
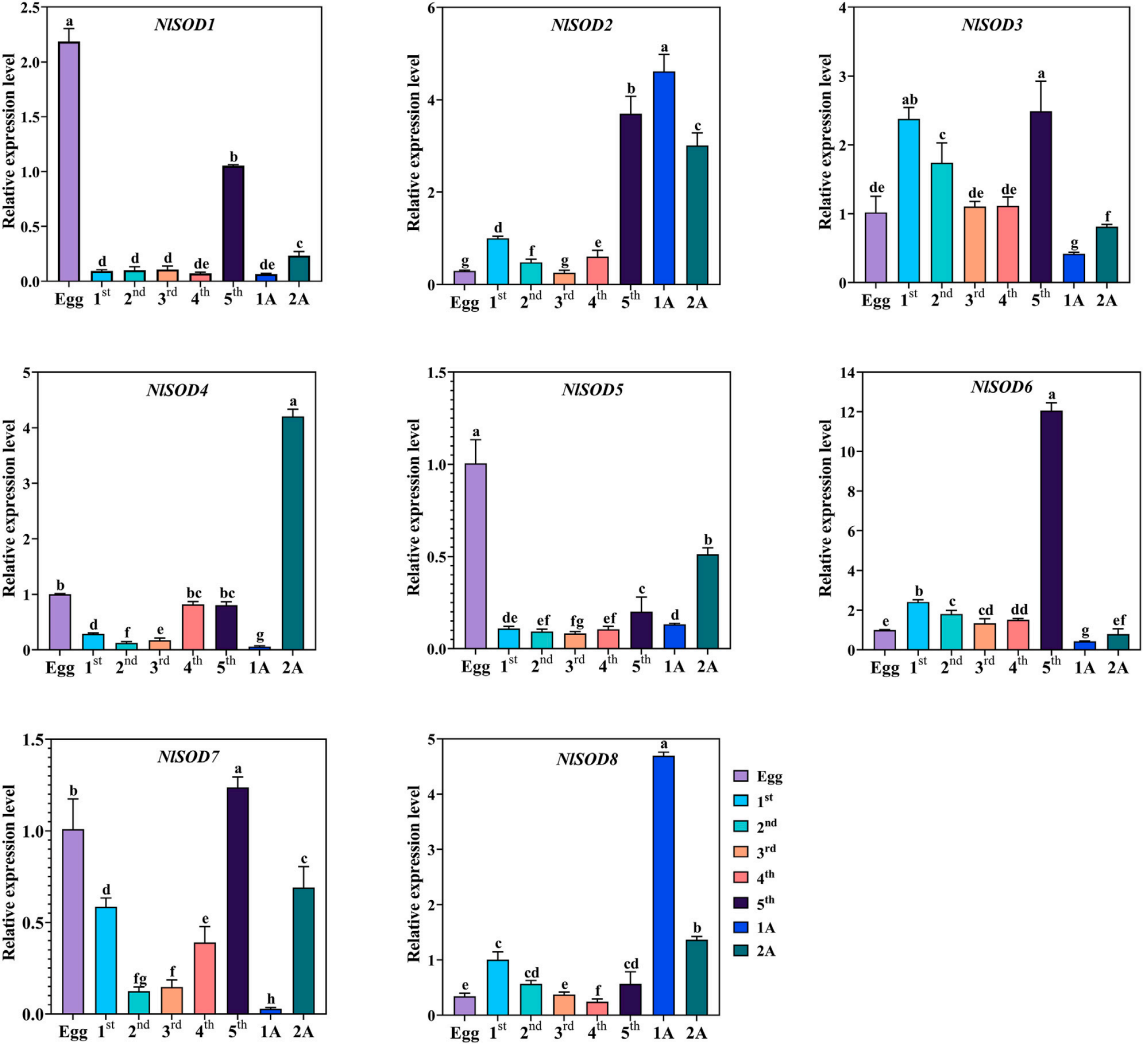
Expression analysis of *NlSOD* genes in developmental stages. 1A represents 1 day after emergence (1 DAE) and 2 DAE. Histogram bars indicate expression, and error bars show means ± SEM. Bars annotated with different lowercase letters are statistically significant at *p* < 0.05 (Tukey test).

Tissue-specific expression analysis unfolded the potential role of *NlSOD* genes in the development of BPH (Figure 4). For instance, dominant expression was observed in the fifth instral stage of *NlSOD6* up to 14 folds, followed by *NlSOD2* in the fifth instral stage, 1 day after emergence (1A) and 2A up to six folds expression. Furthermore, the *NlSOD4* showed moderate transcription in 2A, and *NlSOD8* displayed the five folds expression in 1A. However, the lower transcription was recorded in *NlSOD1*, *NlSOD5,* and *NlSOD7*, suggesting that the *NlSOD* gene family has a potential role in the developmental stages of *N. lugens* (Figure 4). Notably, the lowest transcription was observed by overall *NlSOD* genes in the first to fourth instral stage, excluding *NlSOD3* and *NlSOD7,* which have a moderate expression pattern in the first-fourth instral development stage. Among all the developmental stages, the *NlSOD* genes showed high expression in the fifth instral stage; for a detailed description and a graphical representation, see the appended data in the supplementary file (Supplementary Figure S4).

**FIGURE 5:**
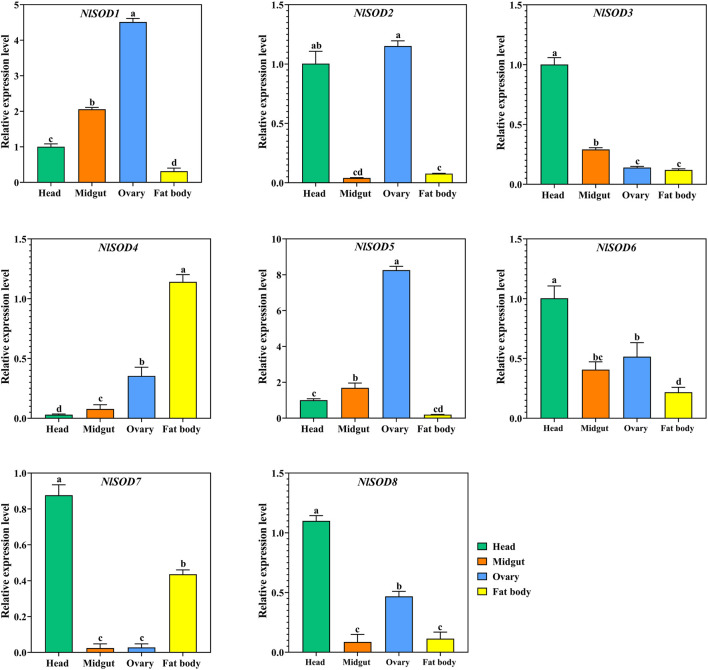
Expression analysis of *NlSOD* genes in selected segments/tissues. Histogram bars indicate expression, and error bars show means ± SEM. Bars annotated with different lowercase letters are statistically significant at *p* < 0.05 (Tukey test).

We have confirmed the tissues specific expression of *NlSOD* genes in four major tissues (head, midgut, ovary, and fat body) at 2 days after emergence (2DAE) by qRT-PCR. The results showed that most of the *NlSOD* genes have the dominant expression mostly in the head and ovary followed by the fat body and midgut (Figure 5). In particular, the high expression of *NlSOD5* up to 10 folds, followed by *NlSOD1* with five folds in the ovary. Moreover, the moderate transcriptional pattern was shown by most of the *NlSOD* genes, such as the *NlSOD2, NlSOD3, NlSOD4, NlSOD6,* and *NlSOD8,* with 1.5 folds in different tissues. A single *NlSOD* family member *NlSOD7* displayed lower transcription in almost all tested tissues (Figure 5).

### 3.6 Expression analysis of the *NlSOD* genes under JGM treatment

**FIGURE 6:**
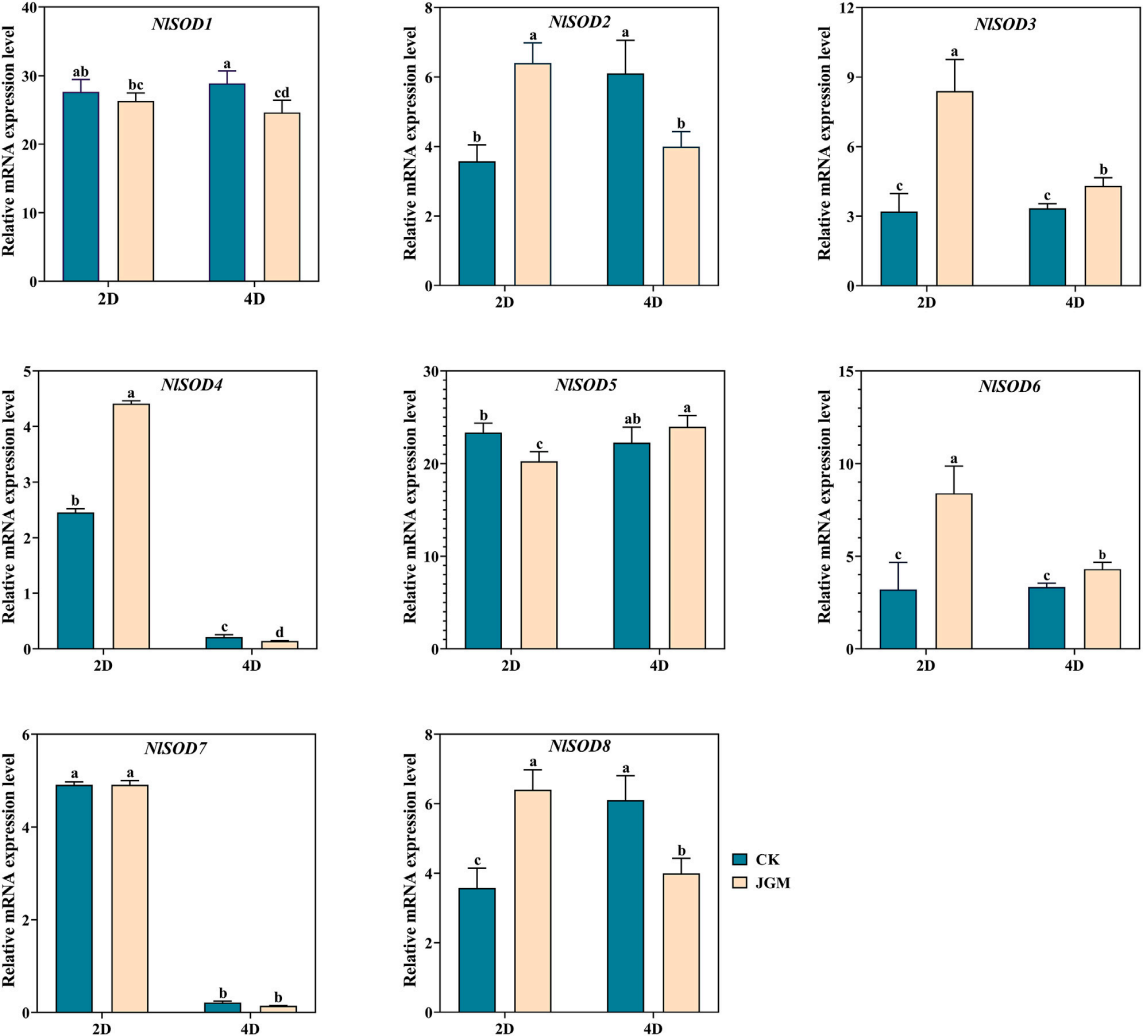
Expression analysis of *NlSOD* genes in response to Jinggangmycin 2 days (2D) and 4D after treatment. The histogram bars indicate expression, and the error bars show means ± SEM. Bars annotated with different lowercase letters are statistically significant at *p* < 0.05 (Tukey test).

We performed qRT-PCR analysis to validate the *NlSOD* genes expressions in response to JGM spraying treatment (Figure 6). Our study revealed that *NlSOD* genes, such as *NlSOD1* and *NlSOD5,* displayed upregulated expressions at 2 days (2D) and 4D time points. Followed by *NlSOD2* and *NlSOD8* had moderate transcription, and the rest of the four genes, such as *NlSOD3, NlSOD4, NlSOD6,* and *NlSOD7,* had the lowest transcription patterns. These results suggest that the JGM spraying application induces the *NlSOD* genes and enhances BPH’s immunity regulations against JGM.

### 3.7 Expression analysis of the *NlSOD* genes in response to glucose, sucrose, and trehalose hydroponic treatment

**FIGURE 7:**
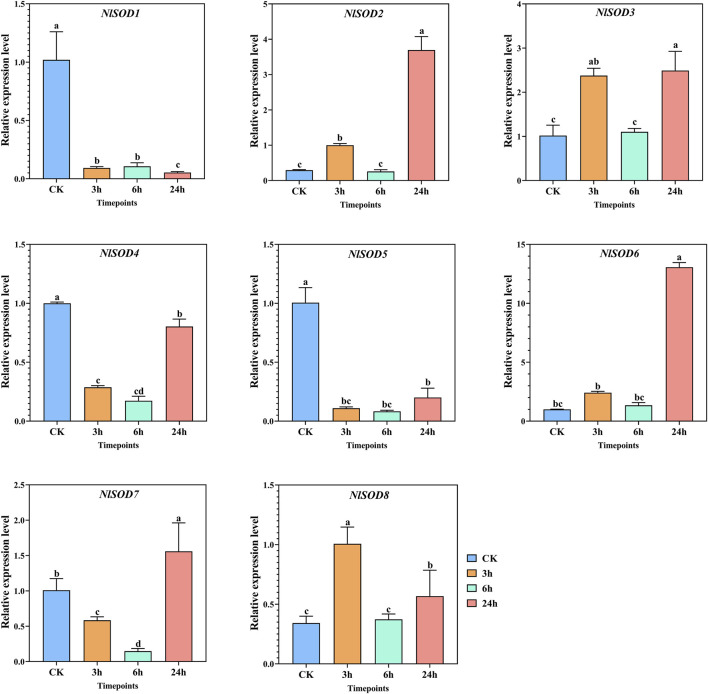
Expression analysis of *NlSOD* genes in response to glucose at 3h, 6h, and 24h. The histogram bars indicate expression, and the error bars show means ± SEM. Bars annotated with different lowercase letters are statistically significant at *p* < 0.05 (Tukey test).

The qRT-PCR analysis revealed the potential role of the *NlSOD* gene family. Among the 8 *NlSOD* genes, the *NlSOD6* gene displayed dominant expression of fifteen folds at 24h, followed by *NlSOD2* and *NlSOD3* with five and four folds expressions under glucose, respectively. Whereas the rest of the genes, such as *NlSOD1*, *NlSOD4, NlSOD5,* and *NlSOD6,* displayed comparatively low transcription at 3 h and 6h, while *NlSOD7* showed a moderate expression of 2.5 folds at 24 h (Figure 7).

**FIGURE 8:**
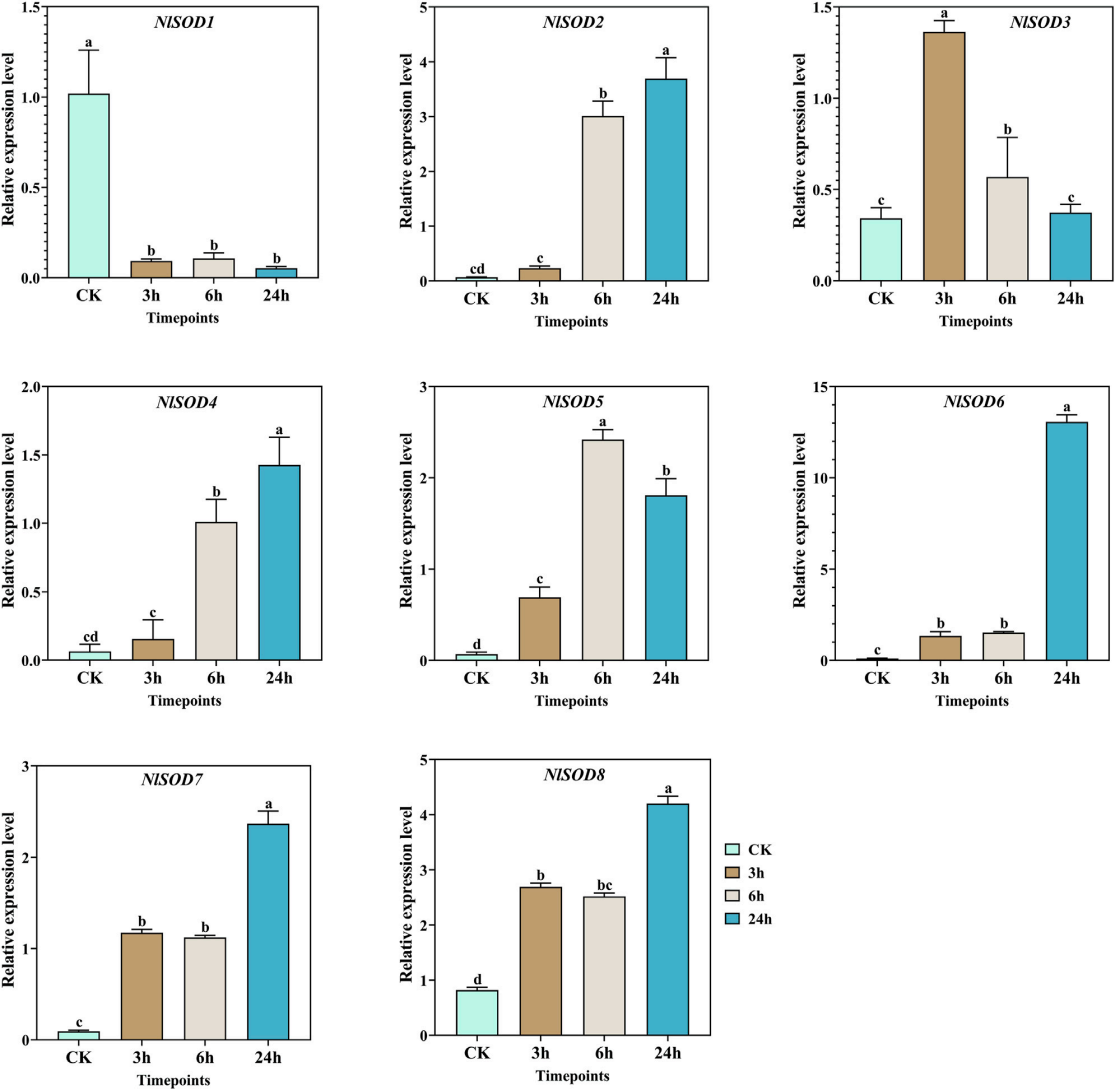
Expression analysis of *NlSOD* genes in response to sucrose at 3h, 6h, and 24h. The histogram bars indicate expression, and the error bars show means ± SEM. Bars annotated with different lowercase letters are statistically significant at *p* < 0.05 (Tukey test).

Sugar transporters may offer a new potential target for controlling insect pests (Kikuta et al., 2010). Herein, the expression of *NlSOD* genes in BPH fed on rice plants treated with sucrose hydroponics was investigated. Similar to glucose, *NlSOD* genes were triggered under sucrose treatment at almost all the time points (Figure 8). Furthermore, the *NlSOD6* displayed a dominant expression of 15 folds at 24 h. Followed by *NlSOD2* and *NlSOD8* of 5 folds expression at 24h, whereas moderate expression was observed by *NlSOD5* and *NlSOD7* of 3 folds at 24h, and the lowest transcription was observed by *NlSOD3* of 1.3 folds at 3 h. Seven of the eight *NlSOD* genes showed an upregulated expression, and only a single gene, such as the *NlSOD1,* displayed a relatively downregulated expression at 3, 6 and 24 h. The results suggested that most of the *NlSOD* genes are highly responsive to sucrose and could work in concert with metabolism to tailor developmental and defense processes.

Gene expression analysis was conducted to understand the role of *NlSOD* genes of BPH fed with rice plants treated with trehalose hydroponic solution*.* Furthermore, the eight *NlSOD* genes showed upregulated expression patterns under trehalose hydroponic treatment. In which the *NlSOD6* was observed with the dominant expression of 15 folds at 24 h. Followed by *NlSOD8* of 6 folds expression at 24h, whereas the four *NlSOD* genes such as *NlSOD1*, *NlSOD2*, *NlSOD4* and *NlSOD7,* showed moderate expression of 5 folds at 24 h. Contrastingly, the *NlSOD5* and *NlSOD3* were observed with the lowest transcription of 4 and 3 folds, respectively. The expression analysis of *NlSOD* genes under trehalose showed upregulated expression patterns and might finetuning the development of BPH and provide defense on a cellular basis.

## 4 Discussion

When insects suffer from environmental stresses such as extreme temperatures and insecticide toxicities, the ROS are spawned (Gao X. L. et al., 2013; Gao X. M. et al., 2013). Metallo enzyme SOD is the most effective intracellular enzymatic antioxidant, ubiquitous in all aerobic organisms and subcellular compartments prone to ROS-mediated oxidative stress. It eliminates extremely toxic ROS, such as O_2_, and reduces the possibility of hydroxyl radical OH generation through a metal-catalyzed reaction of the Haber-Weiss type (Fridovich, 1978). Mn-SOD is considered a general stress-responsive element, and several internal and external cues, including pesticide toxicity at the transcriptional and translational levels, can substantially impact its expression profiles (Zelko et al., 2002; Cho et al., 2006). The SOD enzymes are a group of highly conserved stress response enzymes that function as molecular chaperones assisting in protein refolding and degradation in stressed cells (Mayer and Bukau, 2005; Yamamoto et al., 2005d; Yamamoto et al., 2007). However, only a little is known to date about oxidative stress induced by JGM pesticides and sugars (glucose, sucrose, trehalose) in BPH. In the current study, the *SOD* gene family in BPH was characterized, and the JGM and sugar protective effect of protein was investigated for the first time.

### 4.1 *SOD* genes are widely distributed in class *insecta*

A BLASTP search demonstrated that the *LsSOD* gene family and the *NlSOD* gene family were closely related, with the *LsSOD* genes having a higher proportion of matched sequences, indicating that the *NlSOD* gene is more diverged compared to other *SOD* genes. Moreover, the phylogenetic analysis also confirmed this relationship (Umasuthan et al., 2012). Both insects are members of the same insect family, the Hemiptera (Delphacidae), and share shorter N-terminal sequences, which strongly suggests their taxonomic association (Zelko et al., 2002). The phylogenetic tree analysis demonstrated that Mn-SOD and Cu/Zn-SOD may have shared a common ancestor and that Mn-SOD may have undergone more evolutionary change than Cu/Zn-SOD. Additionally, the Cu/Zn-SOD clade was divided into the Cu/Zn-SOD and Cu/Zn-SOD subgroups (Figure 1), with the latter showing greater divergence; these two protein sub-families may develop through gene replication as previously reported by Liu et al. (2015).

Members of the *SOD* gene family are highly conserved, and our results also uncover this high homology. The current study identified highly inducible *SOD* genes and shared close similarities with other insect species. Comparing the amino acid sequence of NlSOD with its counterparts from Lepidopteran insect species, including *L. striatellus*, *S. incertulas*, *S. frugiperda*, and *D. melanogaster*, the NlSOD showed high similarity with *L. striatellus* (Figure 1).

Domains are a protein’s distinct functional and/or structural units (Marchler-Bauer et al., 2015). Usually, they are responsible for a particular function or interaction, contributing to the overall role of a protein (Sigrist et al., 2010). The current study found that NlSOD proteins exhibit three functional domains (Supplementary Figure S1). The SOD_Fe_N functional domain is reportedly strongly associated with innate immunity, which is essential for survival organisms, for instance, in the survival and stress response in *Chlamys farreri* (Wang et al., 2018). Similarly, we also found the SOD_Fe_N, Sod_Fe_C, and Sod_Cu domains, which might help the BPH withstand the JGM and high sugar.

Additionally, usually, they are responsible for a particular function or interaction; because of these domains in NlSOD proteins, the NlSOD candidate proteins (Supplementary Figure S3) are found in high interactions with other functional proteins. Moreover, sequence motifs are short, recurring patterns in DNA that are presumed to have a biological function. Often, they indicate sequence-specific binding sites for proteins such as nucleases and transcription factors. Others are involved in important processes at the RNA level, including ribosome binding, mRNA processing (splicing, editing, polyadenylation), and transcription termination (D'Haeseleer, 2006). Herein, we also found the five motifs in the *NlSOD* genes (Figure 2) to explain the gene structure and influence their role in development and stress response, which needs further study.

### 4.2 *NLSOD* genes play an essential role in *N. lugens* growth and development

In prior investigations, it was discovered that *SOD* genes were crucial not only for growth and development but also for stress response and other vital processes throughout various tissues and organs (Okado-Matsumoto and Fridovich, 2001; Zelko et al., 2002). In the present study, we found that the *SOD* was also distributed in all the tested tissues, such as in the head, midgut, ovary, and fat body (Figure 5). Additionally, the *SOD* genes showed transcriptional activity in the BPH egg, first to fifth instral nymph, 1 day after emergence (1A) and 2A (Figure 4), suggesting that this transcript may be involved in stress sensing and signaling transduction.

Moreover, there were alterations in the *NlSOD* gene expression levels throughout distinct tissues. However, the fact that the midgut and fat body exhibited the greatest expression implies that it may be contributing to development. The midgut includes the malpighian tubule, which plays an important role in detoxifying and eliminating toxins (Beyenbach et al., 2010). And fat body is one of the prime sites for antioxidant enzymes (Yamamoto et al., 2005b; Lee et al., 2005; Yamamoto et al., 2005). Our findings are consistent with those of *Glossina morsitans* (Munks et al., 2005) and *Agrilus planipennis* (Rajarapu et al., 2011), in which the midgut and fat body showed a substantial upregulation of *SOD* genes. The hindgut and fat body’s upregulation of *SOD* suggests that these two organs served as the primary sites for the production of antioxidant enzymes and provided resistance to oxidative stress.

### 4.3 *NlSOD* genes regulate the BPH immune response to pesticide toxicity and high sugar

**FIGURE 9:**
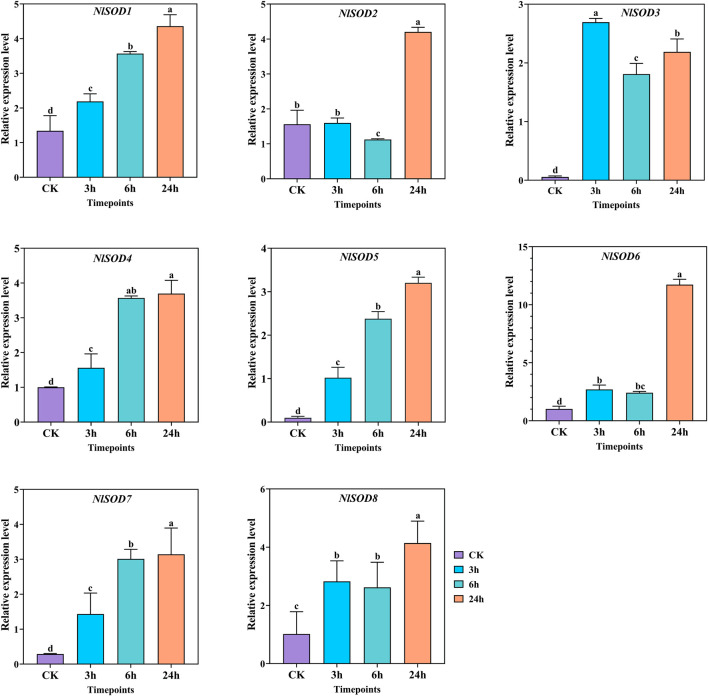
Expression analysis of *NlSOD* genes in response to trehalose at 3h, 6h, and 24h. The histogram bars indicate expression, and the error bars show means ± SEM. Bars annotated with different lowercase letters are statistically significant at *p* < 0.05 (Tukey test).

Aerobic organisms constantly consume oxygen, which the mitochondria utilize to produce the energy required to sustain life (Atalay et al., 2020). Some of these oxygen molecules may convert into reactive oxygen species (ROS) during metabolic processes (Qureshi et al., 2021a). Exposure of tissues and cells to ultraviolet radiation and chemical agents generates ROS such as superoxide anion (O_2_^−^) and hydrogen peroxide (H_2_O_2_) and causes oxidative damage to cells (Hermes-Lima and Zenteno-Savín, 2002; Fukai and Ushio-Fukai, 2011; Lee et al., 2019). *SOD* gene family is an essential antioxidant that catalyzes the dismutation of superoxide anions into molecular O_2_ and H_2_O_2_, thereby protecting cellular components from extreme oxidative stress impairments (Valentine and Hart, 2003b; Fukai and Ushio-Fukai, 2011). Insects have developed tolerance to the insecticides JGM and sugars as a consequence of their widespread application (Matsumura et al., 2008). In the present study, the qRT-PCR analysis showed that most of the *NlSOD* gene expressions were induced after JGM treatment. For instance, the *NlSOD1* and *NlSOD5* displayed dominant expressions (Figure 6). Following that, under the sugar (glucose, sucrose and trehalose) treatment, the *NlSOD6* and *NlSOD2* were observed with high transcription (Figures 7–9) and might be involved in regulating BPH immune response to JGM and high sugar. However, the rest of the *NlSOD* genes displayed mixed expression patterns under the JGM and high sugar treatment and cannot be ruled out from functional studies. These genes may work redundantly and therefore rely on each other transcriptional changes. Interactive protein studies would be useful to confirm the statement of whether these amino acids have homodimeric or heterodimeric interactions. Additionally, our results provide a theoretical platform for pesticide regulatory bodies.

## 5 Conclusion

In the present study, we identified eight *NlSOD* genes from NCBI and classified them into three subgroups based on their functional domain and structural properties. Furthermore, the qRT-PCR analysis revealed that *NlSOD* genes play an essential role in BPH growth and development by expression in all tested tissues. The *NlSOD* genes were also found responsive to JGM and sugar stress. Among them, the *NlSOD6* gene displayed dominant expression and could be involved in regulating BPH response to JGM and sugar. The interactive protein analysis disclosed that these *NlSOD,* in concert with other stress markers, is crucial for BPH response to external stimuli. Stress control is a complex mechanism, and our study demonstrated the potential role of *NlSOD* genes in modulating BPH development. These myriad molecules could be used in future molecular work to manipulate BPH pesticide resistance.

## Data Availability

The original contributions presented in the study are included in the article/Supplementary Material, further inquiries can be directed to the corresponding authors.
