# Supplementary material for: The superoxide dismutase (SOD) genes family mediates the response of Nilaparvata lugens to jinggangmycin and sugar
Source: Front Physiol. 2023 May 10;14:1197395. doi: 10.3389/fphys.2023.1197395 (PMC10228653; doi:10.3389/fphys.2023.1197395)
Supplement: Supplementary file 1 [file DataSheet1.PDF]

The Superoxide dismutase (SOD) genes family induces the response of *Nilaparvata lugens* under Jinggangmycin applications

Ahmad Sheraz[1†], Haowen Zhu[1†], Qiaoqiao Dong[1], Tingting Wang[1], Suman Zong[1], Huaiqi Wang[1], Linquan Ge[1]* and Tao Wu[2]*

† These authors contributed equally to this work.

[1] College of Plant Protection, Yangzhou University, Yangzhou 225009, China.

[2] College of Horticulture and Landscape Architecture, Yangzhou University, Yangzhou 225009

*Correspondence: lqge@yzu.edu.cn; wutao@yzu.edu.cn

Table S1. List of the primers used in the study.

Genes	Primer's sequence 5'-3'
ACTIN	F: TGGACTTCGAGCAGGAAATGG<br>R: ACGTCGCACTTCAGATCGAG
NlSOD1	F: TTTACAGCTTCAGCGAATT<br>R: ACATACCCTCCCAGAACTAC
NlSOD2	F: TTCCAGCGTTGCCAGTTT<br>R: AGGAGCATATCCGACCACC
NlSOD3	F: TTCCGATGACTCCACAAGC<br>R: GCACGGCAAGAACAGCAT
NlSOD4	F: CGCCGTGGAAGTTGAGAAG<br>R: TCACTCTAAGCACCACCAGACA
NlSOD5	F: GCCGTGAGGGTTGAAGTG<br>R: GTCAGCGGTGAAATTAGTGG
NlSOD6	F: GATGTGGAAGCCGTGGTT<br>R: GTGCTGAAGGGTGATGCG
NlSOD7	F: AGACTGAGGGTGCCCGAGGT<br>R: TGCCATTGCCGAGGACTT
NlSOD8	F: GGAGACATGAACGTGGAAGC<br>R: CACCCGATCAACGCAGTAG

Table S2. *NlSOD* genes functional conserved domain analysis (annotations)

Accession Nu	Domain	Annotation
Pfam00081	Sod_Fe_N	SOD catalyzes the conversion of superoxide radicals to hydrogen peroxide and molecular oxygen. Three evolutionarily distinct families of SODs are known, of which the Mn/Fe-binding family is one. In humans, there is a cytoplasmic Cu/Zn SOD and a mitochondrial Mn/Fe SOD. The N-terminal domain is a long alpha antiparallel hairpin. A small fragment of YTRE_LEPBI matches well.
Cl27368	Sod_Fe_C	SOD catalyzes the conversion of superoxide radicals to hydrogen peroxide and molecular oxygen. Three evolutionarily distinct families of SODs are known, of which the Mn/Fe-binding family is one. In humans, there is a cytoplasmic Cu/Zn SOD and a mitochondrial Mn/Fe SOD. The C-terminal domain is a mixed alpha/beta fold.
		SOD catalyzes the conversion of superoxide radicals to hydrogen peroxide and molecular oxygen. Three evolutionarily distinct families of SODs are known, of

Pfam00080	Sod_Cu	which the copper/zinc-binding family is one. Defects in the human SOD1 gene cause familial amyotrophic lateral sclerosis (Lou Gehrig's disease). The structure is an eight-stranded beta-sandwich, like the immunoglobulin fold.

Table S3. Interactive protein partners of NlSOD1 protein.

Target Gene	FlyBase ID	Interactive Partners	Annotation
**NlSOD1**	FBgn0003462	SOD	Superoxide dismutase [Cu-Zn]; Destroys radicals are normally produced within the cells and toxic to biological systems; it Belongs to the Cu-Zn superoxide dismutase family.
	FBgn0010213	SOD2	Superoxide dismutase [Mn], mitochondrial, destroys superoxide anion radicals that are normally produced within the cells and are toxic to biological systems.
	FBpp0080148	Tam	DNA polymerase subunit gamma-1, mitochondrial, is Involved in the replication of mitochondrial DNA. Has both 5'-3' DNA polymerase and 3'-5' exonuclease activity.
	FBpp0074825	Cat	Catalase: Occurs in almost all aerobically respiring organisms and protects cells from the toxic effects of hydrogen peroxide.
	FBgn0033631	SOD3	Extracellular superoxide dismutase [cu-zn]; Superoxide dismutase 3 (Sod3) is an extracellular Cu Zn superoxide dismutase that can be produced as secreted and membrane-bound forms. Sod3 converts oxygen free radicals to hydrogen peroxide and is involved in free radical defense and redox balance; It Belongs to the Cu-Zn superoxide dismutase family.
	FBgn0040319	gclc	Protein binding; glutamate-cysteine ligase activity. It is involved in the biological process described with cellular response to DNA damage stimulus; glutathione metabolic process; glutathione biosynthetic process; It Belongs to the glutamate-cysteine ligase type 3 family.
	FBpp0085780	CG15116	Glutathione peroxidase activity. It is involved in the biological process described with response to oxidative stress, oxidation-reduction process, and multicellular organism reproduction.
	FBpp0084313	CG5948	Superoxide dismutase, cu-zn family; CG5948, isoform A; Zinc ion binding; superoxide dismutase activity; copper ion binding. It is involved in the biological process described with the oxidation-reduction process, removing superoxide radicals.
	FBpp0072932	PHGPx	Peroxidase activity; glutathione peroxidase activity. It is involved in the biological process described with response to lipid hydroperoxide, response to oxidative stress, and oxidation-reduction process.
	FBgn0038570	Prx5	Peroxiredoxin 5, isoform A; Antioxidant activity; oxidoreductase activity. It is involved in the biological process described with negative regulation of innate immune response; response to

			oxidative stress; determination of adult lifespan; negative regulation of apoptotic process; cell redox homeostasis.
	FBgn0034727	mRps29	Mitochondrial ribosomal protein S29; Structural constituent of ribosome. It is involved in the biological process described with translation, the apoptotic process.

In the fifth instar developmental stage, the *NlSOD* genes unfold the potential role by displaying a pulmonated expression pattern (Figure S1). Among the eight *NlSOD* genes, the *NlSOD6* showed a dominant expression of 13.3 folds, followed by *NlSOD2* with a transcription of 3.9 folds. A moderate expression was recorded for *NlSOD3* and *NlSOD7* with expression ranging from 1.5 to 2.5 folds whereas, the *NlSOD1*, *NlSOD4*, and *NlSOD8* displayed an average of one-fold expression, and the lowest transcription was recorded for *NlSOD5*.

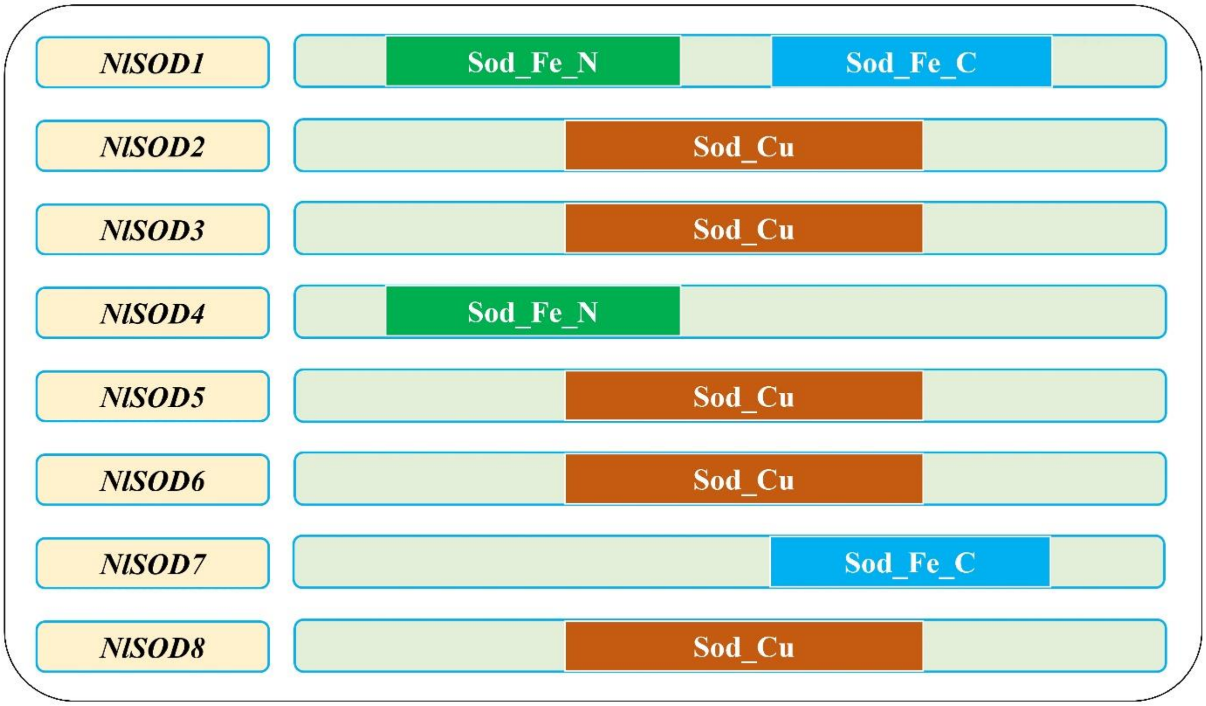

Figure S1. Schematic representation of *NlSOD* conserved domain. The SOD genes domain was identified from the NCBI conserved domain search, and the graphical representation was done using Microsoft PowerPoint 365.

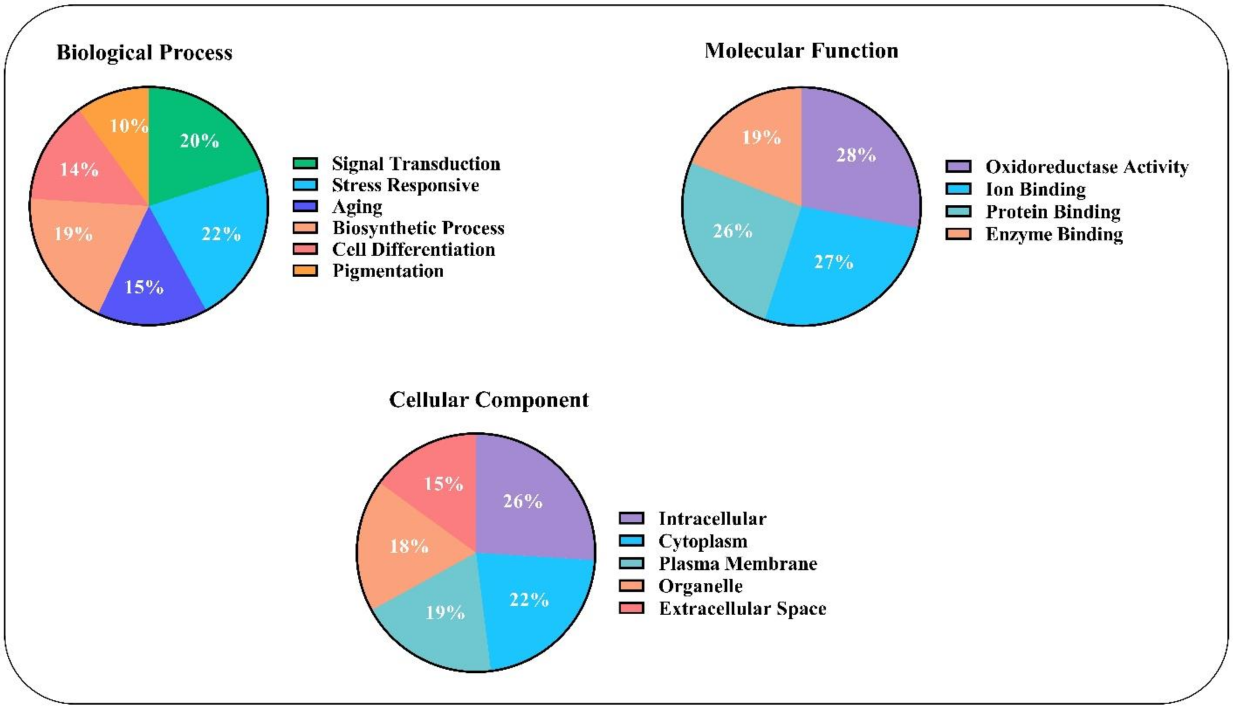

Figure S2. Schematic representation of GO pathways enrichment analysis of *NlSOD* genes. The GO analysis was initially identified using the Cello2Go web server, and from the obtained data, (P-value < 0.05, $\log2 \geq 2, \leq -2$) coregulated JGM stress in comparison with the control group and the final figure was recovered using the GraphPad Prism (Version 9.4.1).

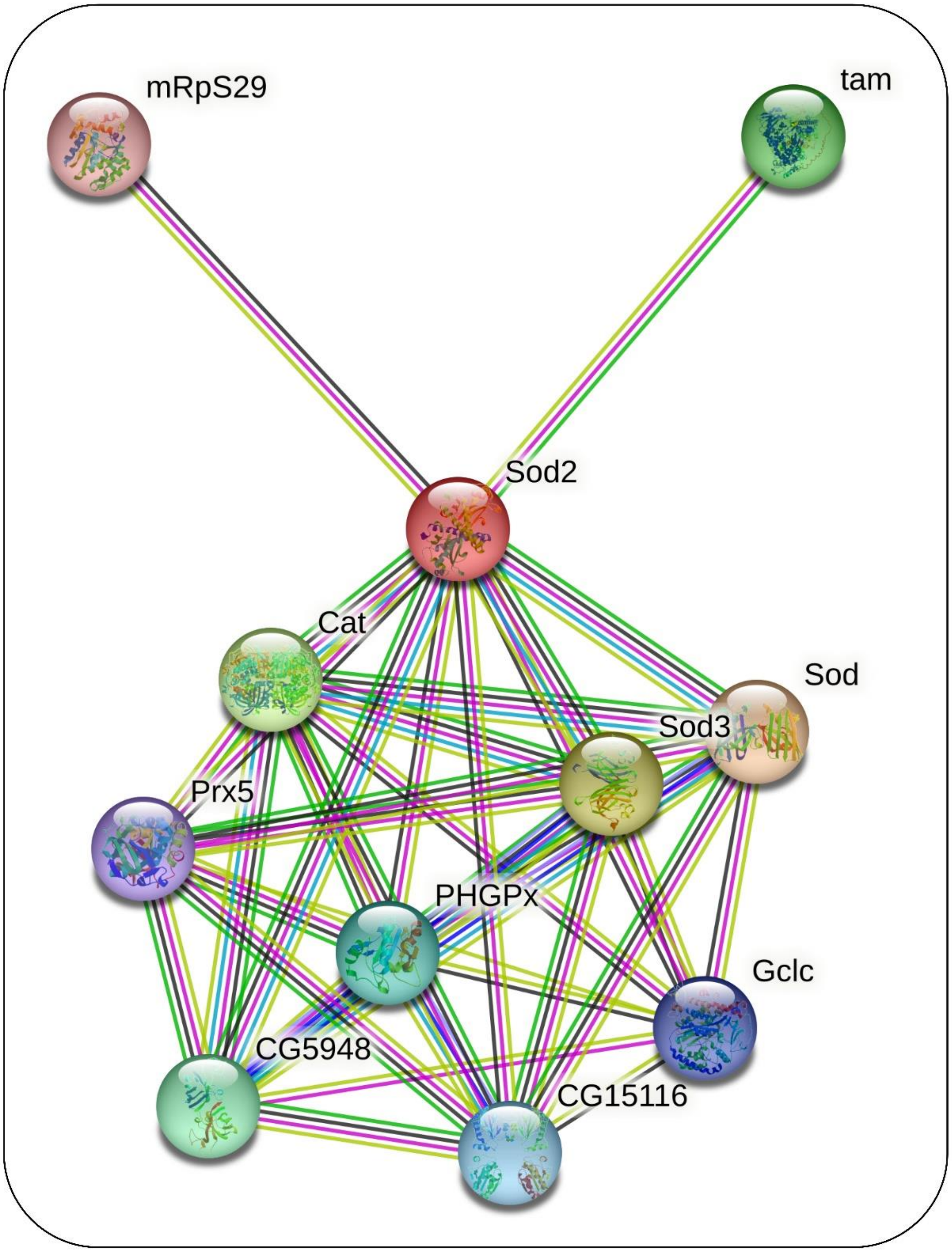

Figure S3. Schematic representation of NlSOD interactive proteins partners. The NlSOD1 protein sequence was searched in the online String server, and the network was recovered.

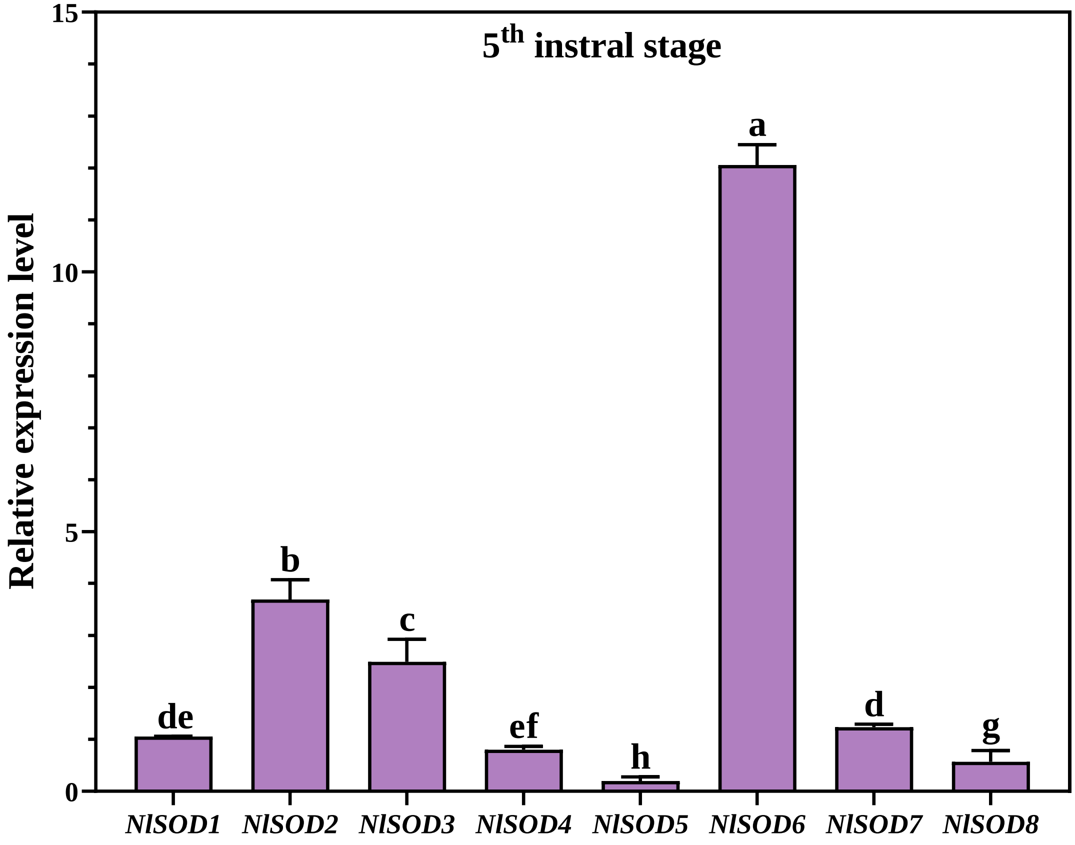

Figure S4. *NlSOD* genes expression in 5th instar developmental stage. Histogram bars indicate expression, and error bars show means ± SEM. Bars annotated with different lowercase letters are statistically significant at $p<0.05$ (Tukey test).
